# Detection of intracellular monosodium urate crystals in gout synovial fluid using optical diffraction tomography

**DOI:** 10.1038/s41598-021-89337-7

**Published:** 2021-05-11

**Authors:** Sangwoo Park, Lucy Eunju Lee, Hanna Kim, Ji Eun Kim, Seung Jun Lee, Sunggyu Yoon, Seungwoo Shin, Heemin Kang, YongKeun Park, Jason Jungsik Song, Seongsoo Lee

**Affiliations:** 1 Division of Aging Research, Gwangju Center, Korea Basic Science Institute (KBSI), 49 Dosicheomdansaneop-ro, Nam-gu, Gwangju, 61751 South Korea; 2 Department of Materials Science and Engineering, Korea University, Seoul, 02841 South Korea; 3 Division of Rheumatology, Department of Internal Medicine, Yonsei University College of Medicine, 50-1 Yonsei-ro, Seodaemun-gu, Seoul, 03722 South Korea; 4 Synapse Center, Yonsei University College of Medicine, Seoul, 03722 South Korea; 5 Department of Physics, Korea Advanced Institute of Science and Technology (KAIST), Daejeon, 34141 South Korea; 6 KAIST Institute for Health Science and Technology, KAIST, Daejeon, 34141 South Korea; 7 Tomocube Inc., Daejeon, 34051 South Korea; 8 Institute for Immunology and Immunological Diseases, Yonsei University College of Medicine, Seoul, 03722 South Korea

**Keywords:** Optical imaging, Gout, Phagocytes

## Abstract

Optical diffraction tomography (ODT) enables imaging of unlabeled intracellular components by measuring the three-dimensional (3D) refractive index (RI). We aimed to detect intracellular monosodium urate (MSU) crystals in synovial leukocytes derived from gout patients using ODT. The 3D RI values of the synthetic MSU crystals, measured by ODT, ranged between 1.383 and 1.440. After adding synthetic MSU crystals to a macrophage, RI tomograms were reconstructed using ODT, and the reconstructed RI tomograms discerned intracellular and extracellular MSU crystals. We observed unlabeled synthetic MSU crystal entry into the cytoplasm of a macrophage through time-lapse imaging. Furthermore, using gout patient-derived synovial leukocytes, we successfully obtained RI tomogram images of intracellular MSU crystals. The 3D RI identification of MSU crystals was verified with birefringence through polarization-sensitive ODT measurements. Together, our results provide evidence that this novel ODT can identify birefringent MSU crystals in synovial leukocytes of patients with gout.

## Introduction

Gout is the most common cause of inflammatory arthritis due to the deposition of monosodium urate (MSU) crystals, and the disease has an increased worldwide prevalence^1^. The detection of MSU crystals by polarized light microscopy is the gold standard for gout diagnosis because it is fast, reliable, and irreplaceable due to their strong birefringence^2^. Sometimes differentiation between MSU and other crystals such as calcium pyrophosphate (CPP) crystals can be challenging due to weak birefringence and small size of CPP crystals, especially when only a few crystals are present in synovial fluid^3^. Detection of intracellular crystal is important essential in diagnosing crystal-induced arthritis because extracellular crystal can be detected as incidental finding or contamination. Therefore, further characterization of intracellular crystals by optical diffraction tomography (ODT) can provide additional information for the diagnosis as well as disease mechanism study of crystal-induced arthritis.

ODT is a three-dimensional (3D) microscopic technique that enables reconstructing the 3D refractive index (RI) distribution of a microscopic object in a label-free and non-invasive manner^4^. By measuring the 3D RI, ODT can visualize the intracellular components of live cells. ODT has been recently applied in diverse fields to monitor label-free morphological changes in live cells during drug treatment^5^, disease progression^6^, nanoparticle tracking^7^, and photodynamic therapy^8^. However, it has not yet been used as a diagnostic tool to evaluate clinical samples despite its usefulness.

In this proof-of-concept study, we attempted to obtain the 3D RI tomograms of MSU crystals and assess the potential of this novel imaging modality to detect MSU crystals for the diagnosis of gouty arthritis. By exploiting their distinct RI values, MSU crystals were imaged and distinguished from other biological structures. Furthermore, by exploiting the superior 3D and temporal resolution of ODT, assessing the interaction between MSU crystals and synovial mononuclear cells was conducted. Using a polarizer-analyzer pair, the birefringence of MSU crystals was evaluated using polarization-sensitive ODT measurements in synovial fluids of patients with gout. To the best of our knowledge, this is the first attempt to apply 3D ODT technology in MSU detection in clinical samples.

## Results

### Visualizing intracellular synthetic MSU crystals in macrophages by ODT

**Figure 1:**
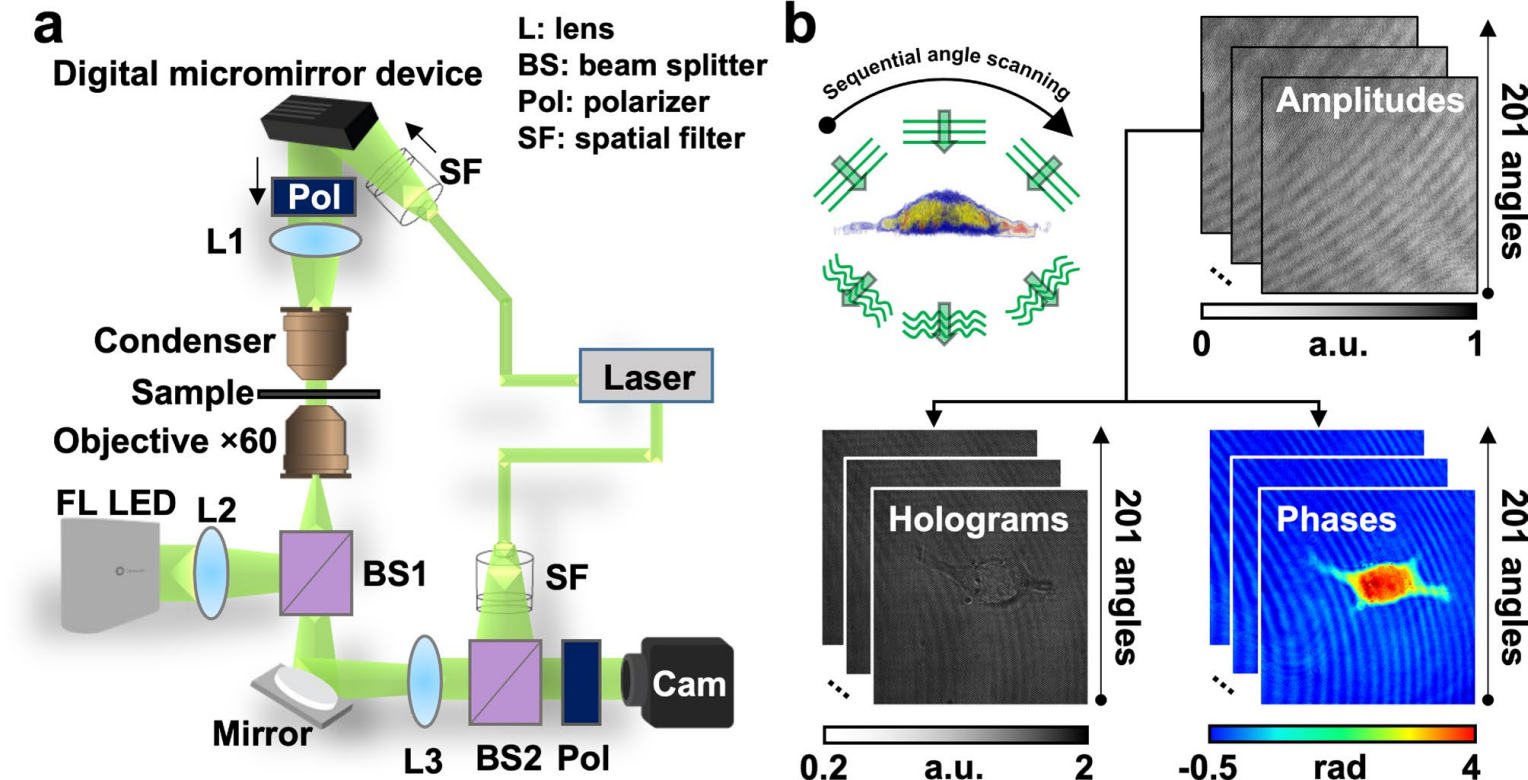
Schematic diagrams of three-dimensional (3D) optical diffraction tomography (ODT) microscopy. (**a**) Optical assembly of an ODT microscope. (**b**) A representative sample cell is shown. Label-free ODT produces a refractive index (RI) distribution by assembling the RI range of images acquired from 201 sequential angles scanned from two-dimensional holograms.

The ODT apparatus used in this study is illustrated in Fig. 1a. Through Mach–Zehnder interferometric microscopy equipped with a digital micromirror device (DMD), multiple 2D holograms of a sample were acquired from varying illumination angles. The diffracted beam from a sample interferes with a tiled reference beam and generates spatially modulated holograms. The recorded holograms, amplitude, and phase images diffracted from a sample were obtained using the phase retrieval algorithm (Fig. 1b)^5,9,10^. Using ODT principles, a 3D RI tomogram of a sample was reconstructed from multiple 2D holograms.

**Figure 2:**
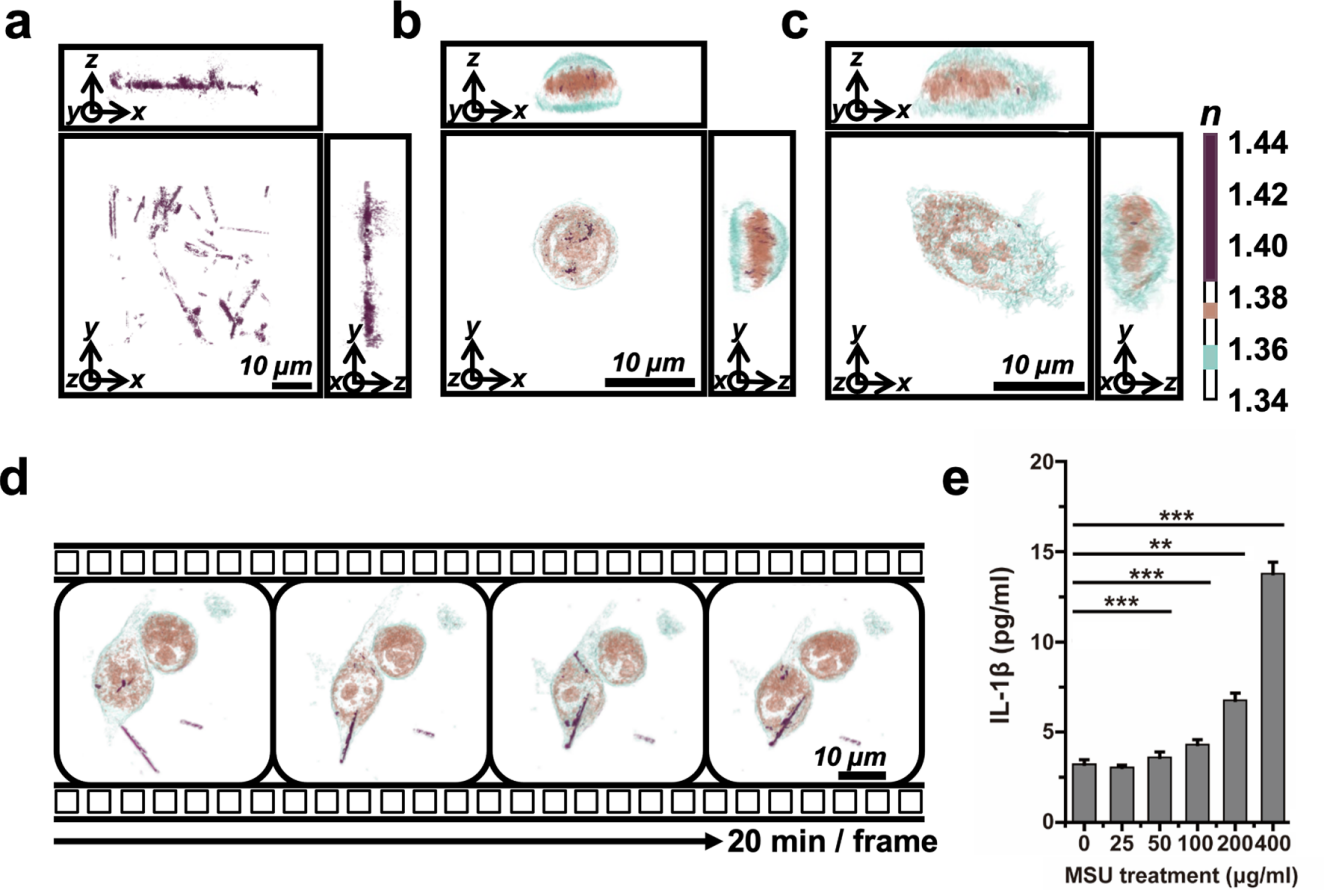
Three-dimensional (3D) optical diffraction tomography (ODT) of monosodium urate (MSU) crystals. (**a**) Synthetic MSU crystals were recorded as RI ≥ 1.385. See also Supplementary Movie S1. (**b**) A monocytic THP-1 cell. (**c**) A macrophage derived from a THP-1 cell after treatment with phorbol 12-myristate 13-acetate (PMA) for 72 h. The color bar indicates the 3D rendered refractive index (RI) distribution (n = 1.340–1.440). Each square shows the x–y, y–z, or x–z plane. n: refractive index. (**d**) Time-lapse monitoring of macrophage THP-1 cells after treatment with 10 µg/mL monosodium urate (MSU) crystals for 2 h. The MSU crystals, with RI ≥ 1.385, are indicated in purple. See also Supplementary Movie S2. (**e**) IL-1β production of THP1 cells assessed via ELISA at 3 h after treatment with MSU crystals. **P < 0.01, ***P < 0.001, ns: not significant.

To determine whether 3D ODT can detect intracellular MSU crystals, we first assessed the morphology of synthetic MSU crystals by measuring the RI. The MSU crystals appeared as randomly distributed microrod structures with a broad range of RI, from 1.383 to 1.440, in cell culture medium (Fig. 2a and Supplementary Movie S1). To investigate the 3D morphological changes in human macrophages, we determined the RI of THP-1 cells through real-time monitoring using 3D ODT. The 3D RI distribution of monocytic THP-1 cells revealed spherical suspended cells with distinct nuclear membranes (Fig. 2b). Upon administration of PMA, monocytic THP-1 cells differentiated into macrophages, and they were extended after adhesion (Fig. 2c). The average volume of THP-1 cells was increased to 1.74-fold from 1634.5 to 2842.6 µm^3^ when activated (Supplementary Fig. S1). We also investigated MSU crystal phagocytosis in real-time by monitoring their RI under label-free conditions. Representative images, obtained every 20 min for 2 h, showed single cells with clearly discernible intracellular MSU crystals of higher RI range compared to those of cellular structures (Fig. 2d). Upon treatment with MSU crystals, the crystals permeated THP-1 cells, and the cellular morphology was maintained for 2 h (Supplementary Movie S2). These results demonstrated that MSU crystals could interact with human THP-1 macrophages and result in morphological changes in the cells. To confirm the inflammasome activation in THP-1 cells by MSU crystals, we assessed the expression of IL-1β at 3 h after the exposure to MSU crystals. IL-1β production was significantly induced by MSU crystals in a dose-dependent manner at 3 h (Fig. 2e). We also investigated intracellular MSU crystal detection by 3D ODT with murine macrophage RAW 264.7 cells with similar findings (Supplementary Fig. S2, Supplementary Movie S4 and S5).

### Cell–crystal interaction in the synovial fluid of patients with gout

**Figure 3:**
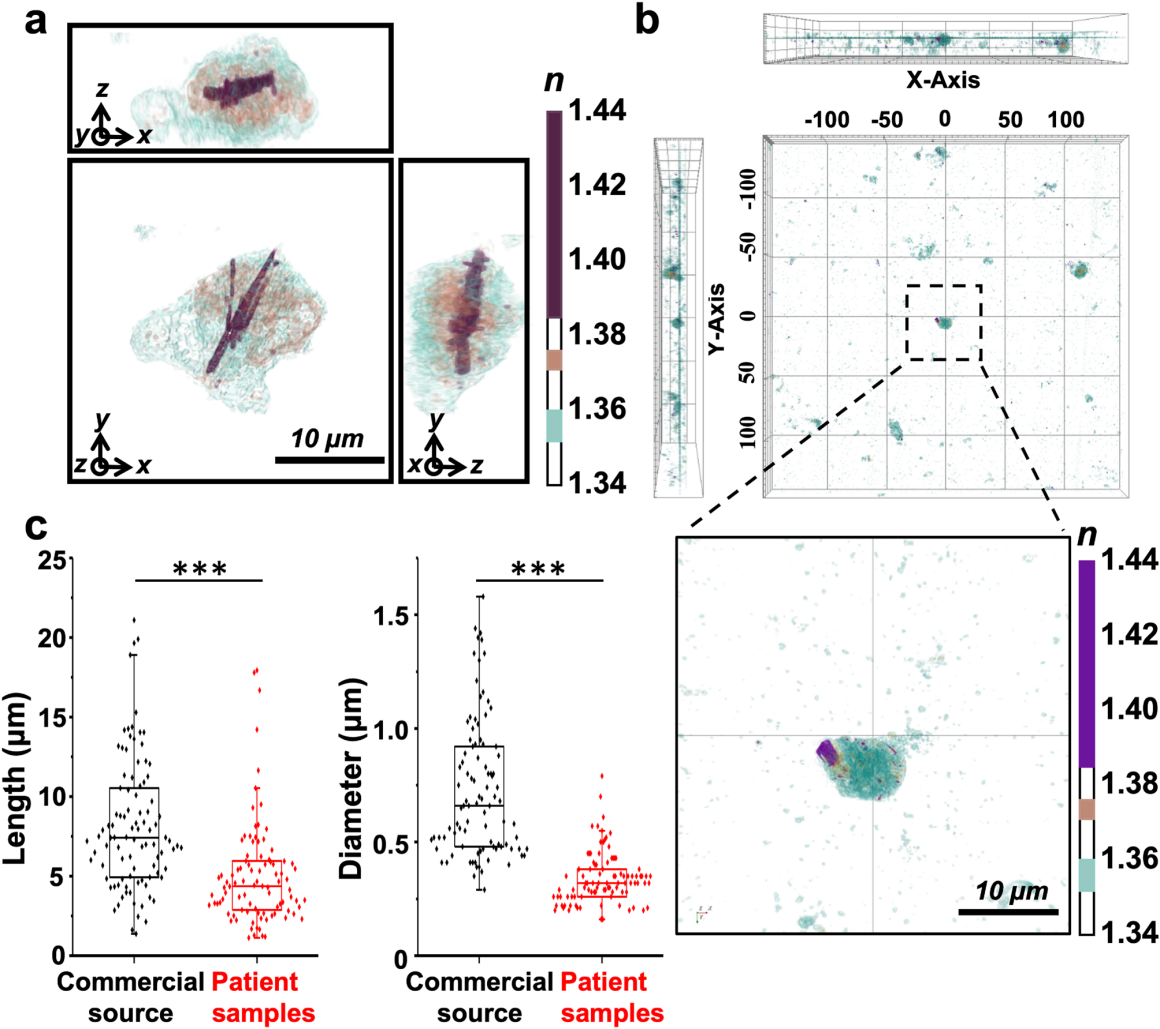
Monosodium urate (MSU) crystals acquired from patients with gout were compared with synthetic MSU crystals. (**a**) Three-dimensional (3D) holotomographic image of MSU crystals embedded in the patient cells. The color bar indicates the 3D rendered RI distribution range (n = 1.340–1.440). Each square shows the x–y, y–z, or x–z plane. n: refractive index. Additionally, see Supplementary Movie S3. (**b**) Extensive monitoring of MSU crystals from a synovial fluid sample through stitch mode of holotomographic imaging (300 µm × 300 µm). The boxed region is enlarged in the lower panel. (**c**) Comparison of patient crystals with commercial MSU crystals (n = 96). Data are represented as the mean ± SD values. ***P < 0.001.

We examined the applicability of ODT for the diagnosis of gouty arthritis by detecting MSU crystals in the synovial fluid of patients with gout. The morphological evaluation demonstrated that intracellular MSU crystals from gout patients displayed microrod structures and had higher RI than other cellular structures (Fig. 3a and Supplementary Movie S3). Furthermore, ODT could easily detect MSU crystals from a wide area (300 µm × 300 µm) using stitch mode of holotomographic imaging (Fig. 3b). We subdivided a wide region of interest for synovial leukocytes into multiple smaller sections, and each section was imaged and combined by stitching to produce a larger overview. In the boxed region, images of cells from the synovial fluid were visually enlarged to precisely confirm the presence of intracellular MSU crystals (Fig. 3b bottom image). In this highly magnified image, the MSU crystals in the RI region are pseudocolored purple. In addition, we quantitatively compared the synthetic MSU crystals from a commercial source and MSU crystals from the synovial fluid of patients with gout. The lengths of commercially prepared synthetic MSU crystals and MSU crystals from gout patients were 8.19 ± 4.13 µm and 5.49 ± 4.48 µm, and their diameters were 0.74 ± 0.32 µm and 0.34 ± 0.11 µm, respectively. These results indicate that MSU crystals in the synovial fluid of a patient with gout were shorter and thinner than commercial MSU crystals.

### Validation of 3D RI identification of MSU crystals by polarization-sensitive ODT measurements

**Figure 4:**
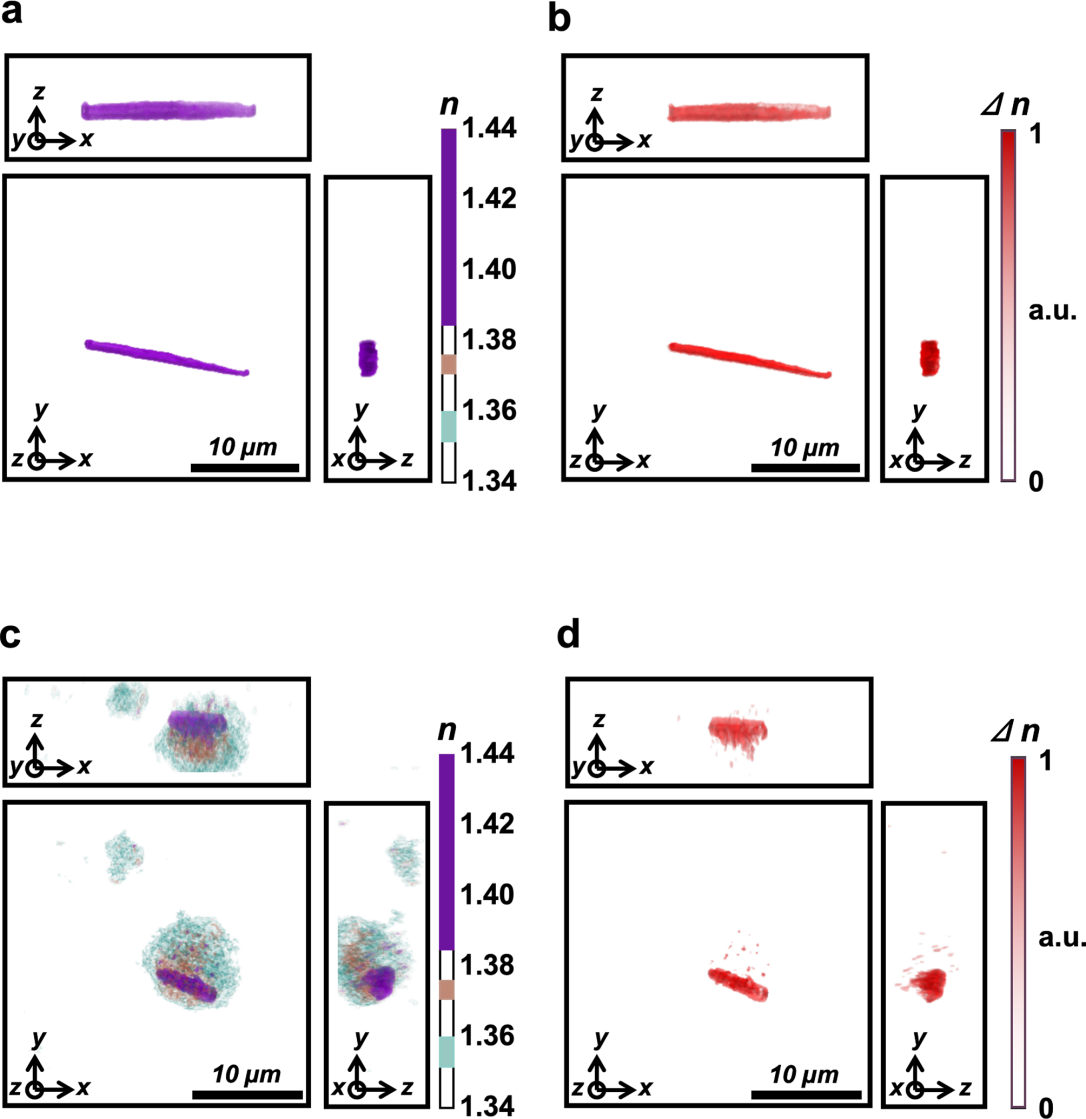
Birefringence properties of patient monosodium urate (MSU) crystals and polarization-sensitive RI contrast tomogram. (**a**) A three-dimensional (3D) holotomographic image of synthetic MSU crystals under a polarized light source with a 0° angle. (**b**) Polarization contrast image of MSU crystals under a polarizing filter used during optical diffraction tomography (ODT) with a 90° angle. (**c**) 3D holotomographic image of synovial mononuclear cells. Intracellular MSU crystal is distinguished by its relatively higher RI value, as shown in previous figures. (**d**) Polarization contrast image of MSU crystal-containing synovial mononuclear cells, obtained under a polarizing filter in ODT with a 90° angle. In this image, all structures without birefringence disappeared, and the remaining MSU crystal was shown in red color. n: refractive index, ∆n: change of refractive index under the two different polarizing filter angles.

For validation of the present experiment setup, the birefringence of MSU crystals was also studied. Due to its birefringence, MSU crystals show 3D morphology differing from isotropic objects, including medium, cytoplasm, and other subcellular organelles. Baseline 3D ODT images of synthetic MSU crystals at 0° of the polarization filter were acquired (Fig. 4a), followed by images acquired at 90°. Polarization contrast images were generated by calculating the difference between the two, which demonstrated the birefringence of the synthetic MSU crystals (Fig. 4b). Similarly, 3D ODT images of synovial mononuclear cells from patients with gout were observed using a polarizing microscope at 0° of the polarization filter (Fig. 4c). The polarization contrast images created by the difference in 3D ODT images between 90° and 0° of the polarization filter demonstrated the birefringence of intracellular MSU crystals from clinical samples (Fig. 4d). Other cellular components were invisible because they did not have birefringent properties. Thus, a comparison of distinct RI tomograms (Fig. 4a,c) with polarization-sensitive RI tomograms (Fig. 4b,d) resulted in successful 3D RI identification of intracellular MSU crystals.

## Discussion

ODT is a 3D quantitative phase-imaging modality to determine the RI of a microscopic sample and reconstructs its 3D spatial RI distribution. Since an MSU crystal has a homogeneous RI throughout its structure, ODT could be a suitable imaging modality to visualize these crystals and distinguish them from other structures in biological samples. This study shows that upon imaging with ODT, the MSU crystals were discernible inside or outside cells, indicating that ODT can be used to diagnose gouty arthritis.

Reconstructed RI images of the crystals reproduced a 3D structural counterpart of their conventional microscopic images to visualize their characteristic needle-shaped body, given the specific RI distribution throughout their structures. Since the median length of MSU crystals is a few micrometers, the submicrometric resolution capability of the ODT enabled the reconstruction of the crystal structures such that they were observed as individual rod-shaped 3D objects. Since leukocytes from the synovial fluid play an important role in initiating inflammatory responses in acute gout^11^, we cultured a human macrophage cell line to evaluate macrophages and MSU crystals interaction. Consistent with the results of previous studies on the applications of ODT to live-cell imaging^12,13^, macrophages could be identified from the specific morphology of their plasma membrane and intracellular structures based on RI measurements. The MSU crystals were recognizable extracellularly among macrophages and even within the cells because of their specific RI and shape. Owing to the 3D property of this novel imaging modality, the spatial relationship between a portion of the crystal and a cell can be precisely described as being inside the cell or on the cell surface. Consequently, the morphological differences between the cells with or without the crystals could be identified. Macrophages penetrated by a sharp segment of MSU crystals had their cytoplasm protruding along the remaining extracellular region of the crystal, and they displayed changes in their morphology. They had a more irregular form when penetrated by MSU crystals than their resting, relatively spherical form.

The key advantage of ODT is that it facilitates the assessment of the morphology of the differentiated macrophages and their phagocytic function due to its ability to obtain time-course images of live cells without labeling agents that could disrupt the cellular physiology. We recorded the initial process of phagocytosis as a series of time-course images upon crystal exposure and internalization^14^. Initially, the macrophages interacted with the pointed edge of the MSU crystals, and the attached plasma membrane was actively rearranged to engulf the crystal within 2 h, which is comparable to the reported engulfment time of other particles^15^. To the best of our knowledge, this is the first time a 3D video recording was obtained of phagocytosis of MSU crystals by human macrophages without the use of labeling or staining methods. To demonstrate the biological significance of this process, we also verified the crystal-induced inflammasome activation by showing the increased IL-1β production after crystal phagocytosis^16^.

ODT was performed using samples from patients with gout. In the synovial fluids of patients with gout, we identified synovial mononuclear cells with irregular morphology and a relatively large cytoplasmic volume, wherein multiple crystals were internalized and entrapped. The RI image could be reconstructed based on previously obtained RI information from MSU crystals and THP-1 cells since the RI of MSU crystals from patients did not significantly differ from those of synthetic MSU crystals. This 3D high-resolution imaging modality suggests ODT can be applied for gout diagnosis. Additionally, ODT can scan a large area up to 8 × 8 mm using the latest ODT and software technology in stitch mode. Consequently, ODT is a favorable candidate tool for image-based diagnosis of gouty arthritis, and it can easily be automated.

Another intriguing application is polarization contrast ODT^17^. Since the ODT system can determine the RI of the samples, birefringent material, including MSU crystals, can be specifically detected through changes in their RI through a polarized light source of the ODT system. We assessed this hypothesis by adding a polarizing filter to our microscope. As expected, birefringent MSU crystals were unequivocally observed by their distinctive RI under polarized light. When this method was used to image patient samples, we could only obtain an image of the MSU crystals by subtracting two images under the different polarization conditions. This result indicated the potential of ODT to detect the birefringence of MSU crystals and revealed that ODT could be applied directly for the diagnosis of gouty arthritis.

Overall, this study describes the potential of ODT in the diagnosis of gout, but it has limitations. Firstly, since we assessed only MSU crystals, and no ODT data is available for other crystals, it cannot be concluded that ODT can distinguish MSU crystals from other types of crystals, such as CPP crystals. The determination of the RI values of different crystals is needed to verify the specificity and increase the diagnostic potential of ODT. Secondly, the specificity and sensitivity of ODT for the detection of MSU crystals should be evaluated with larger sample size. Thirdly, it cannot be ruled out that some MSU crystals from synovial fluids of gout patients may have RI values outside the studied range. Future studies should carry out imaging from more clinical samples, including diverse cell types and different crystals in crystal-induced arthropathies.

In conclusion, ODT is a compelling novel imaging modality that can be a potential diagnostic tool for gout since it can measure the RI, a distinctive optical property of MSU crystals. Considering its diverse advantages, including its 3D nature, non-invasiveness, and temporal monitoring potential, ODT is an attractive method as a research tool for cell-crystal interaction and phagocytosis. Furthermore, as an efficient and objective diagnostic tool, ODT data can be easily implemented in an automated system and machine learning algorithms. Therefore, ODT is potentially a suitable candidate diagnostic tool for the automated detection of MSU crystals.

## Methods

### Cell culture: cell lines, media, and induction of differentiation

The human THP-1 monocytic cell line (THP1-HMGB1-Lucia, InvivoGen, San Diego, CA, USA) was used. THP-1 cells were incubated in a T-75 flask containing Roswell Park Memorial Institute 1640 Medium (RPMI 1640; Gibco, Grand Island, NY, USA) supplemented with 10% fetal bovine serum, 1% penicillin, 1% streptomycin, and 1 mM HEPES buffer (Welgene) at 37 °C, in a humidified 5% CO_2_ incubator. Suspended monocytic THP-1 cells were harvested by centrifugation for 3 min at 300×*g*. The supernatant was discarded, and the resuspended cells were incubated for 3 days in a T-75 flask. For ODT measurement, monocytic THP-1 cells were cultured at 1 × 10^5^ cells/mL in 3 mL RPMI media in a specialized culture dish (TomoDish, Tomocube, Daejeon, Korea). For differentiation into macrophages, the THP-1 cells on the TomoDish were sub-cultured in a culture medium with 100 ng/mL of phorbol 12-myristate 13-acetate (PMA; Sigma-Aldrich, St. Louis, MO, USA), 100 µg/mL of Normocin (InvivoGen), and 100 µg/mL of Zeocin (Invitrogen, Carlsbad, CA, USA) for 72 h^18,19^. The cells were cultured in a stage-top humidified incubator (TomoChamber, Tomocube) at 37 °C. The THP-1 macrophage cells on TomoDish were monitored with various concentrations of synthetic MSU crystals (InvivoGen) using ODT (HT-2H, Tomocube).

### 3D RI image acquisition using ODT

3-D cellular morphologies based on RI distribution were acquired using a commercial holotomographic microscope incorporating a Mach–Zehnder interferometer setup (HT-2H, Tomocube), based on a previously reported ODT protocol^5,20^. A coherent laser (λ = 532 nm) was divided into the sample and reference beams. The laser was systematically steered by a DMD for sequential illumination angle scanning^21^. The light scattered by a sample passes through an objective lens (60 ×) and transmitted to the camera to interfere with the reference beam. From the recorded series of interference patterns, the scattered light’s phase and amplitude can be retrieved and processed to reconstruct the 3D RI map based on the previously proposed algorithm^5,9,10^. According to the acquired RI information, the cells were rendered in 3D, using built-in software (TomoStudio, Tomocube). Total image acquisition time is less than 2 min, including seconds for RI measurement and less than a minute for 3D rendering. The length and diameter of MSU crystals were measured from the reconstructed 3D images using TomoStudio. A broad region of RI images was acquired using a stitch mode of ODT to obtain high-resolution images of clinical samples with a large field of view. Multiple tiled RI tomograms were measured from the lateral translation of a sample, and these numerous tiled RI tomograms were stitched into one tomogram with an extended field of view.

### Polarization-sensitive ODT measurements

To validate the 3D RI identification of MSU crystals in cells, polarization-sensitive ODT was used. For the polarization-sensitive ODT measurements, a polarizer-analyzer pair was used as a polarization filter, and 3D RI tomograms were measured twice in different orthogonal directions of the polarizer. Consequently, the difference between the two RI tomograms obtained with orthogonal polarization states of the illumination beam provides polarization-sensitive RI tomograms, which allows MSU crystals to be distinguished because of their birefringence.

### Quantification of IL-1β

Monocytic THP-1 cells were cultured in 96-well tissue culture plates at 1 × 10^5^ cells/well in the presence of 25 ng/mL of PMA for 48 h at 37 °C in a humidified 5% CO_2_ atmosphere. THP-1 macrophages were treated with 0, 25, 50, 100, 200, and 400 μg/mL of synthetic MSU crystals, and supernatants from each sample were collected after 3 h of incubation. The level of IL-1β was measured by enzyme-linked immunosorbent assay (ELISA) using an IL-1β human ELISA kit (Invitrogen) per the manufacturer’s protocol. The optical density signal was measured using the FlexStation 3 microplate reader (Molecular Devices, San Jose, CA, USA).

### Collection of human synovial fluid from patients with gout

Synovial fluids were obtained from knee joints during acute inflammation for diagnosis of joint pain. After negative birefringent MSU crystals were confirmed by polarized light microscopy, the remaining synovial fluid was centrifuged at 430×*g* for 10 min, and the pellet was resuspended in cell freezing media, 90% fetal bovine serum (Gibco) and 10% DMSO (Amresco, Solon, OH, USA), at 1 × 10^5^ cells/mL. The sample was maintained in liquid nitrogen storage at − 80 °C until analysis. At the time of analysis, each sample was thawed by placing the cryovial in a 37 °C water bath, washed with phosphate-buffered saline three times, and resuspended in RPMI media at 1 × 10^3^ cells/mL. The sample (1 mL) was placed in a TomoDish and imaged using ODT. Experiments were approved by the Institutional Review Board (IRB) of human subjects at Severance Hospital (IRB no. 4-2019-0846) and were conducted according to the IRB guidelines and regulations and the Declaration of Helsinki. Written informed consent was obtained from all patients.

### Statistical analysis

Statistical analysis was conducted using Origin software (OriginLab, Northampton, MA, USA). Data are presented as mean ± standard deviation (SD). We performed a Mann–Whitney test to analyze the statistical differences between each group. *P* < 0.05 was set as the level of significance.

## Supplementary Information

Supplementary Information 1.

Supplementary Video S1.

Supplementary Video S2.

Supplementary Video S3.

Supplementary Video S4.

Supplementary Video S5.

Supplementary Figure S1.

Supplementary Figure S2.

## Data Availability

All data generated or analyzed during this study are included in this published article and its supplementary information files.
