# Supplementary material for: Detection of intracellular monosodium urate crystals in gout synovial fluid using optical diffraction tomography
Source: Sci Rep. 2021 May 11;11:10019. doi: 10.1038/s41598-021-89337-7 (PMC8113554; doi:10.1038/s41598-021-89337-7)
Supplement: Supplementary file 1 — Supplementary Information 1. [file 41598_2021_89337_MOESM1_ESM.pdf]

Detection of intracellular monosodium urate crystals in gout synovial fluid using optical diffraction tomography

Sangwoo Park[1,2#], Lucy Eunju Lee[3#], Hanna Kim[3], Ji Eun Kim[3], Seung Jun Lee[3,4], Sunggyu Yoon[1], Seungwoo Shin[5], Heemin Kang[2], YongKeun Park[5,6,7], Jason Jungsik Song[3,8*], Seongsoo Lee[1*]

[1]Division of Aging Research, Gwangju Center, Korea Basic Science Institute (KBSI), Gwangju 61751, Korea

[2]Department of Materials Science and Engineering, Korea University, Seoul 02841, Korea

[3]Division of Rheumatology, Department of Internal Medicine, Yonsei University College of Medicine, Seoul 03722, Korea

[4]Synapse Center, Yonsei University College of Medicine, Seoul 03722, Korea

[5]Department of Physics, Korea Advanced Institute of Science and Technology (KAIST), Daejeon 34141, Korea

[6]KAIST Institute for Health Science and Technology, KAIST, Daejeon 34141, Korea

[7]Tomocube Inc., Daejeon 34051, Korea

[8]Institute for Immunology and Immunological Diseases, Yonsei University College of Medicine, Seoul 03722, Korea

[#] These authors contributed equally to this work.

[*] These authors jointly supervised this work. Correspondence and requests for materials should be addressed to S.L. (e-mail: soolee@kbsi.re.kr) and J. J. S. (e-mail: JSKSONG@yuhs.ac)

Address correspondence to:

Jason Jungsik Song, M.D., Ph.D.

Division of Rheumatology, Department of Internal Medicine, Yonsei University College of Medicine

50-1 Yonsei-ro, Seodaemun-gu, Seoul 03722, South Korea

Tel: +82-2-2228-1930, Fax: +82-2-393-6884, E-mail: JSKSONG@yuhs.ac

ORCID: 0000-0003-0662-7704

Seongsoo Lee, Ph.D.

Division of Aging Research, Gwangju Center, Korea Basic Science Institute (KBSI)

49 Dosicheomdansaneop-ro, Nam-gu, Gwangju 61751, South Korea

Tel: +82-62-712-4419, Fax: +82-62-530-0519, E-mail: soolee@kbsi.re.kr

ORCID: 0000-0002-8012-9749

Supplementary information

Supplementary Movie S1. 3D morphological structure of monosodium urate (MSU) crystals.

Supplementary Movie S2. Time-lapse monitoring of macrophagic THP-1 cells with MSU crystals (10 μg/mL). The MSU crystals with RI ≥ 1.385 are indicated in purple.

Supplementary Movie S3. 3D holotomographic image of synovial leukocytes. The MSU crystals with RI ≥ 1.385 are indicated in purple.

Supplementary Movie S4. 3D morphological structure of RAW264.7 cells with MSU crystals (200 μg/mL). The MSU crystals with RI ≥ 1.385 are indicated in purple.

Supplementary Movie S5. Time-lapse monitoring of RAW264.7 cells with MSU crystals (10 μg/mL). The MSU crystals with RI ≥ 1.385 are indicated in purple.

Supplementary Figure S1. Cellular volume of THP-1 cells with or without phorbol 12-myristate 13-acetate (PMA). Data are shown as the mean ± SEM.

Supplementary Figure S2. Three-dimensional (3D) optical diffraction tomography (ODT) of murine RAW 264.7 cells with or without MSU crystals. (**a**) 3D RI distribution of RAW 264.7 cells. (**b**) RAW 264.7 cells treated with 50 μg/mL MSU crystals. (**c**) RAW 264.7 cells treated with 200 μg/mL MSU crystals. Each square shows the x-y, y-z, or x-z plane. Additionally, refer to Supplementary Movies S4–S5. n: refractive index.

Supplementary methods

Culture of RAW 264.7 macrophage cell line

The murine RAW 264.7 macrophage cell line (TIB-71, ATCC, Manassas, VA, USA) was used. RAW 264.7 cells were cultured in Dulbecco's Modified Eagle's Medium (Welgene, Gyeongsan, Korea) supplemented with 10% fetal bovine serum, 1% penicillin, and 1% streptomycin at 37 °C in a humidified 5% CO_2 incubator. Thereafter, 1×10^5 RAW 264.7 cells were sub-cultured in a specialized

culture dish (TomoDish, Tomocube, Daejeon, Korea) and incubated in a stage-top humidified incubator (TomoChamber, Tomocube) at 37 °C in a humidified 5% CO_2 incubator. The incubated RAW 264.7 cells were monitored before (**Supplementary Figure S2a**) and after treatment with 50 μg/mL (**Supplementary Figure S2b**) or 100 μg/mL (**Supplementary Figure S2c**) of synthetic MSU crystals (InvivoGen) using ODT (HT-2H, Tomocube) (**Supplementary Movie S4** and **S5**).
