# Supplementary figures and images for: Detection of intracellular monosodium urate crystals in gout synovial fluid using optical diffraction tomography
Source: Sci Rep. 2021 May 11;11:10019. doi: 10.1038/s41598-021-89337-7 (PMC8113554; doi:10.1038/s41598-021-89337-7)

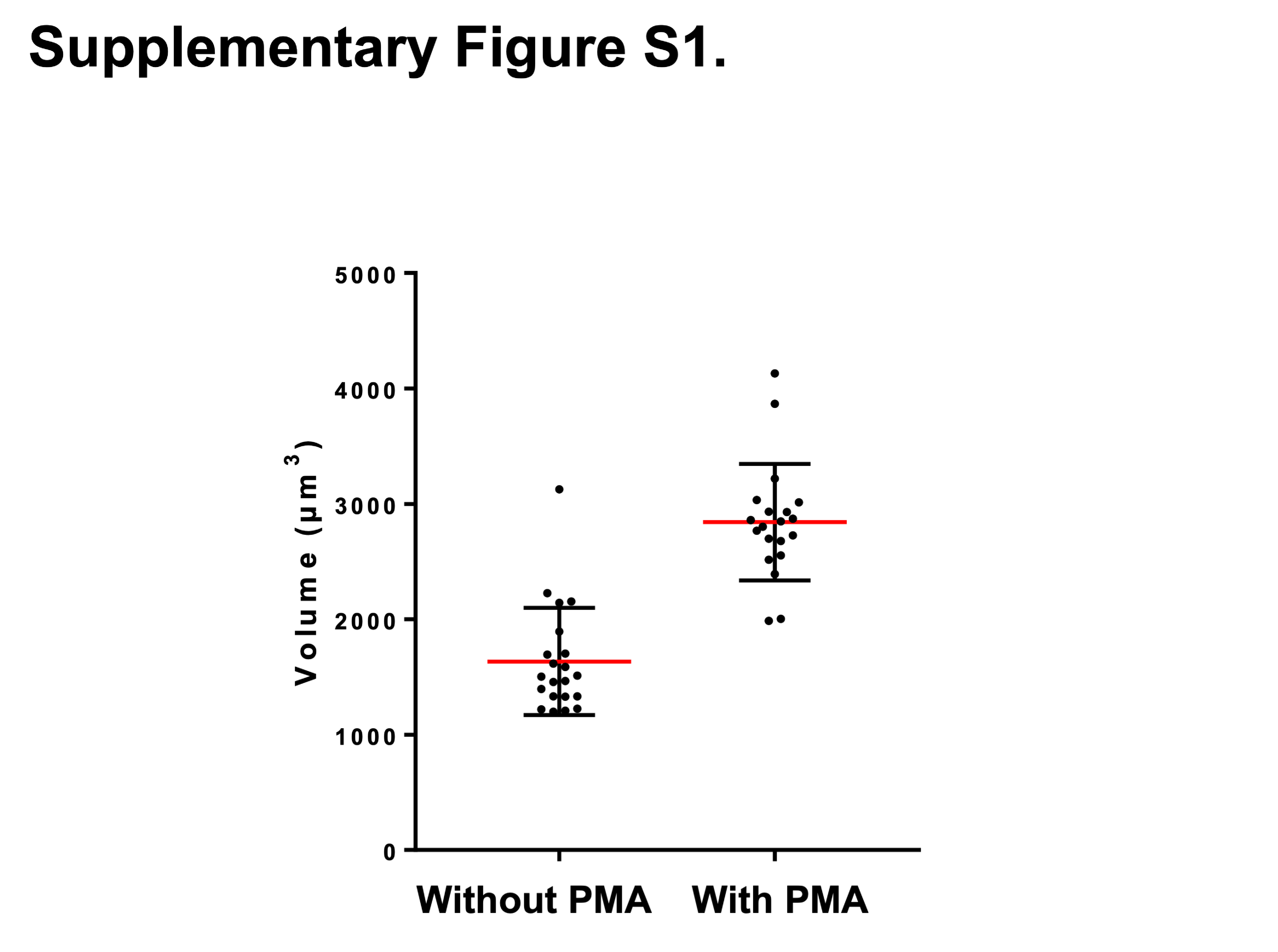

Supplement: Supplementary file 7 — Supplementary Figure S1. [file 41598_2021_89337_MOESM7_ESM.tif]

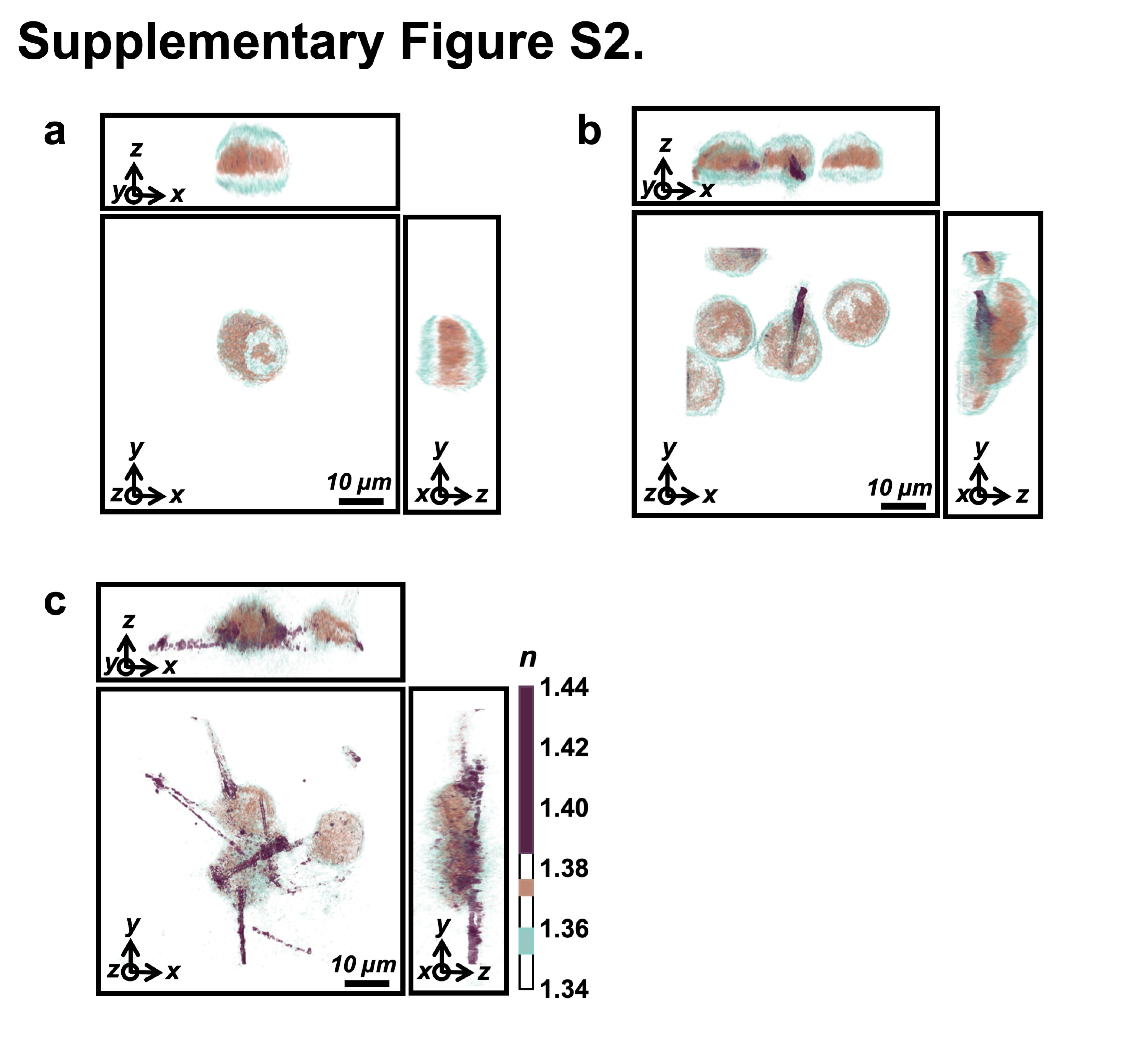

Supplement: Supplementary file 8 — Supplementary Figure S2. [file 41598_2021_89337_MOESM8_ESM.tif]
